# Coordination Analysis Reveals Differences in Motor Strategies for the High Bar Longswing among Novice Adults

**DOI:** 10.1371/journal.pone.0067491

**Published:** 2013-06-26

**Authors:** Albert Busquets, Michel Marina, Alfredo Irurtia, Rosa M. Angulo-Barroso

**Affiliations:** 1 Institut Nacional d’Educació Física de Catalunya, Universitat de Barcelona, Barcelona, Spain; 2 Center for Human Growth and Development & School of Kinesiology, University of Michigan, Ann Arbor, Michigan, United States of America; VU University Amsterdam, The Netherlands

## Abstract

Coordination between arm-trunk and trunk-leg is important for effective longswing performance. This research describes inter-segmental coordination changes after a practice period of longswing on high bar in a novice cohort. Novices were divided by initial skill level (talent) into two groups: spontaneously-talented, (ST, n = 10, closer to expert performance) and non-spontaneously-talented (NST, n = 15). Additionally, post-practice longswing coordination was compared to expert gymnasts (n = 9). Longswing amplitude and coordination (inter-joint reversal points and continuous relative phase, CRP) were assessed for pre- and post-practice sessions. ANOVAs showed similar practice effects in swing enlargements for the ST (11%) and NST (18%), but inter-joint reversal points and positive area in CRP during the downswing were different. Due to practice, the ST group paired shoulder and hip reversal points (events) during the downswing closer and with larger velocity of the arm in relation to the trunk than the NST group. The NST failed to modify coordination probably due to a large variability at the beginning of the downswing. Given a similar amount of practice, talent could help to achieve the right temporal events’ sequence during downswing, which would allow the exploration of different segmental coordination. However, upswing coordination of the novice groups (ST and NST) requires more focused practice to achieve expert levels than downswing, especially the arm-trunk coordination.

## Introduction

The longswing is a task in which gymnasts go from handstand to handstand position through rotation around the high bar with a straight body [1]. The coaching literature has identified the ‘regular’ or traditional backward longswing as a key skill in gymnastics [1], [2], [3]. The longswing is acquired by gymnasts during long periods of practice. Gymnasts start performing longswings with small amplitudes. With training, swings increase progressively in amplitude, finally performing complete longswings from handstand to handstand.

In addition, several studies emphasize the importance of the hip and shoulder flexion and extension for the successful execution of the longswing [1], [4], [5]. Two key functional characteristics of “good high bar longswing mechanics” have been identified by Irwin and Kerwin [2], [3]: (1) hip functional phase, a rapid hyper-extension to flexion of the hip after the gymnast passes through the lowest part of the movement (vertical under the bar) and (2) shoulder functional phase, hyper-flexion to extension of the shoulder joint before the highest point of the circle (vertical over the bar). Preparative actions, preceding the hip and shoulder functional phases, can be defined on the bases of coaching and scientific literature [5], [6]. During downswing the hip moves from flexion to hyper-extension preparing the hip functional phase, while the shoulder changes from extension to hyper-flexion to prepare the shoulder functional phase.

Adequate hip and shoulder coordination are necessary to achieve good performance at both functional phases and their preparative actions. Traditionally, researchers assessed coordination observing the spatio-temporal relation between segments [7], [8]. Coordination between trunk and thigh segments can inform about hip movements, while shoulder actions can be best described by the segmental arm-trunk coordination. Such coordination is one of the main concerns an individual must resolve when faced with a novel task [9], [10,]. Changes in coordination mode emerge from the interplay between the constraints imposed on the degrees of freedom of the system associated with the individual, the task, and the environment [11], [12]. The specific circumstances affect the impact of these three categories of constraints (individual, task, and environment) on the mode of coordination. Practice is considered an important factor within individual constraints that can induce permanent improvements in the ability to perform a motor skill [13], [14]. In fact, expert performance can only be obtained after thousands of hours of practice [15], [16].

While the amount and quality of practice individuals may accumulate will highly impact movement acquisition, spontaneous talent or a-priori talent should also be taken into consideration [17], [18]. In this paper, we focused on changes in coordination and defined talent as the individual’s capacity to be successful to perform a longswing due to some developmental advantage (i.e., an individual or organismic constraint). Improvements in motor skill are therefore related with the individual’s pre-existing capacities making the analysis of this initial state of the system a key point for understanding motor acquisition [19]. In fact, different effects of practice in the performance of lateral swings (i.e., in the frontal plane) on a suspended platform were found in participants who did not possess the same skill level at the beginning of practice [20]. These authors suggested that these differences could arise from individual-specific organismic constraints.

Complexity of a motor skill can be summarized using a single parameter [21], [22], [23]. Changes in this single parameter (i.e., skill global index) entail changes in other underlying skill relevant parameters (i.e., motor strategies). As a result, improvements in the global index require more efficient motor strategies. To examine changes in motor strategies, one can focus on performance, that is, changes in movement outcomes; or on the spatio-temporal relationships between body parts (coordination) [19], [23]. For example, in the longswing, performance would refer to when within the swing cycle the maximal hip flexion occurs, while coordination would refer to the spatio-temporal relationship between the thigh and the trunk. Several studies suggested that performance and coordination acquisition occur in parallel but with different time rate during a motor skill acquisition [24], [25]. Two stages were proposed: (1) early learners’ focus on the appropriate placement of task events (i.e., performance) to achieve functional and goal-directed movements; and (2) later in skilled performance, the major focus will be the dynamic control of the action (i.e., coordination). Following this proposal, it would be possible to find changes in the global index and performance but not in coordination when motor acquisition is assessed in early learners.

A chance to assess motor strategy changes (i.e., performance and coordination) is the acquisition of a new sport skill. Longswing on high bar performance has been investigated in previous research to evaluate its optimal kinematics and kinetics characteristics [1], [4], [5], [26]. While improvements in performance and perception due to practice and initial skill level (i.e., a-priori talent) in gymnastics have been previously examined [27], practice effects on the body limbs coordination executing a longswing on high bar considering the a-priori talent have not been contemplated in previous studies. While a single parameter (skill global index) to assess changes at the level of overall outcome seems to be appropriate to examine practice effects, the selection of the coordination variables should take into consideration the congruency between these coordinative variables, functional phases, and practical application.

The primary aim of this research was to quantitatively describe movement coordination changes after a 9-weeks practice period of a task (longswing on the high bar) in a novice cohort that was divided in two groups based on initial level of the skill global index and performance (i.e., events placement): spontaneously-talented versus non-spontaneously-talented. For the purpose of this study and given the task characteristics, the skill global index of the longswing was characterized by the longswing amplitude [28], and we focused on the inter-segmental coordination between arm-trunk and trunk-thigh to provide more congruency between functional phases, and practical application. Spontaneous talent may be an important discriminatory factor of the individual that affects changes in skill acquisition. We assumed that when the early attempts of novices without previous experience are quantitatively closer to those of experts, and that this spontaneous talent proves to further improve the placement of relevant longswing events (i.e., performance) after the same amount of practice [27], then the spontaneously-talented group may have a greater potential to improve movement coordination among the relevant segments involved in these events. Following suggestions presented in previous studies [13], [19], [20], we hypothesized that, within the novice cohort and after similar content and practice amount, those subjects with spontaneous talent for this task will experience further improvements in coordination than those less talented. In contrast, improvements in skill global index will be similar in both groups. As a secondary aim, novices’ coordination was compared to expert gymnasts at post-practice to assess whether the changes in the novice groups were in a direction approaching experts (i.e., improvements).

## Methods

### Participants

Twenty-five students (fifteen males and ten females, age 20.2±2.2 years; height  =  1.70±0.07 m; body mass  =  66.5±11.7 kg) formed the novice cohort. All of them practiced sports activities, but none had experience with swinging on high bar’s gymnastics. The 25 participants were classified using a k-means Cluster (Systat 11.0, Systat Software, Inc., San José, CA, USA) analysis into two skill groups: non-spontaneously-talented (NST, 8 males and 7 females, age 20.0±2.0 years; height  =  1.70±0.07 m; body mass  =  66.5±13.4 kg) and spontaneously-talented (ST, 7 males and 3 females, age 20.7±2.6 years; height  =  1.71±0.08 m; body mass  =  66.4±9.2 kg). Cluster analyses were computed on the basis of the longswing’s skill global index and performance variables (i.e., longswing amplitude and events and phases variables, respectively; see *Task events* section in Method) from their best executed longswing during the first bout in the first practice session. In this study also participated an additional expert group (E) consisting of nine gymnasts from the national team (6 males and 3 females, age 19.0±4.5 years; height  =  1.59±0.13 m; body mass  =  54.9±15.3 kg), who had more than five years of competition experience and averaged ten training sessions per week. Therefore, they accumulated thousands of hours of training allowing them to perform the longswing at the top level [15], [16]. All participants were fit and injury free and each signed a consent form to participate in the study. The study was approved by the Ethic Committee of Clinic Researches of the Catalan Sport Administration.

### Experimental protocol

The experiment was carried out in a gymnasium on a regular high bar. Training straps and a plastic tube were used to attach the participants’ hands to the bar to reduce emotional distress, increase security, and avoid blisters. For consistency, experts also used the straps and tube. At the starting position, the participants were suspended quiet and in an extended position under the bar. From the starting position without any preparatory actions, participants were asked to increase longswing amplitude in ten successive longswings per bout in order to achieve the maximal amplitude of their swings. Longswings were performed following standards defined by the Fédération Internationale de Gymnastique (FIG) Code of Points [29] (i.e., full extension of arms and legs).

The task was practiced for 20 minutes during 18 sessions per participant (two sessions per week). Nine weeks of practice is the estimated time by expert coaches to learn the longswing in novice adults. At the beginning of the first session an expert gymnastics coach, blind to novice group membership, taught participants how to perform a longswing on the high bar through graphical and verbal explanation of the events and phases. In addition, the coach proceeded to demonstrate the skill. The participants were requested to perform ten longswings per bout. An average of five bouts of ten longswings per session was performed by each participant. A new bout only started if the participant deemed him/herself completely recovered avoiding fatigue. The expert gymnastics coach provided standard verbal feedback about performance errors during the execution of repetitions and at the end of the bout.

### Data collection and reduction

Data were collected for the first bout during session 1 and the last bout during session 18, and the longswing with the largest amplitude of these first and last bouts were selected qualitatively by the expert coach for analysis. Two digital video cameras (Handycam DCR-HC23E Mini DV, SONY, Japan) recorded the movements at 50 Hz. Cameras were located at 1.37 m height, one in each side of the plane containing the bar, and forming a 90° angle between their optical axes. A reference system was defined with the *y* axis as the high bar, the vertical axis as the *z*, and the axis perpendicular to this plane as the *x*.

**Figure 1:**
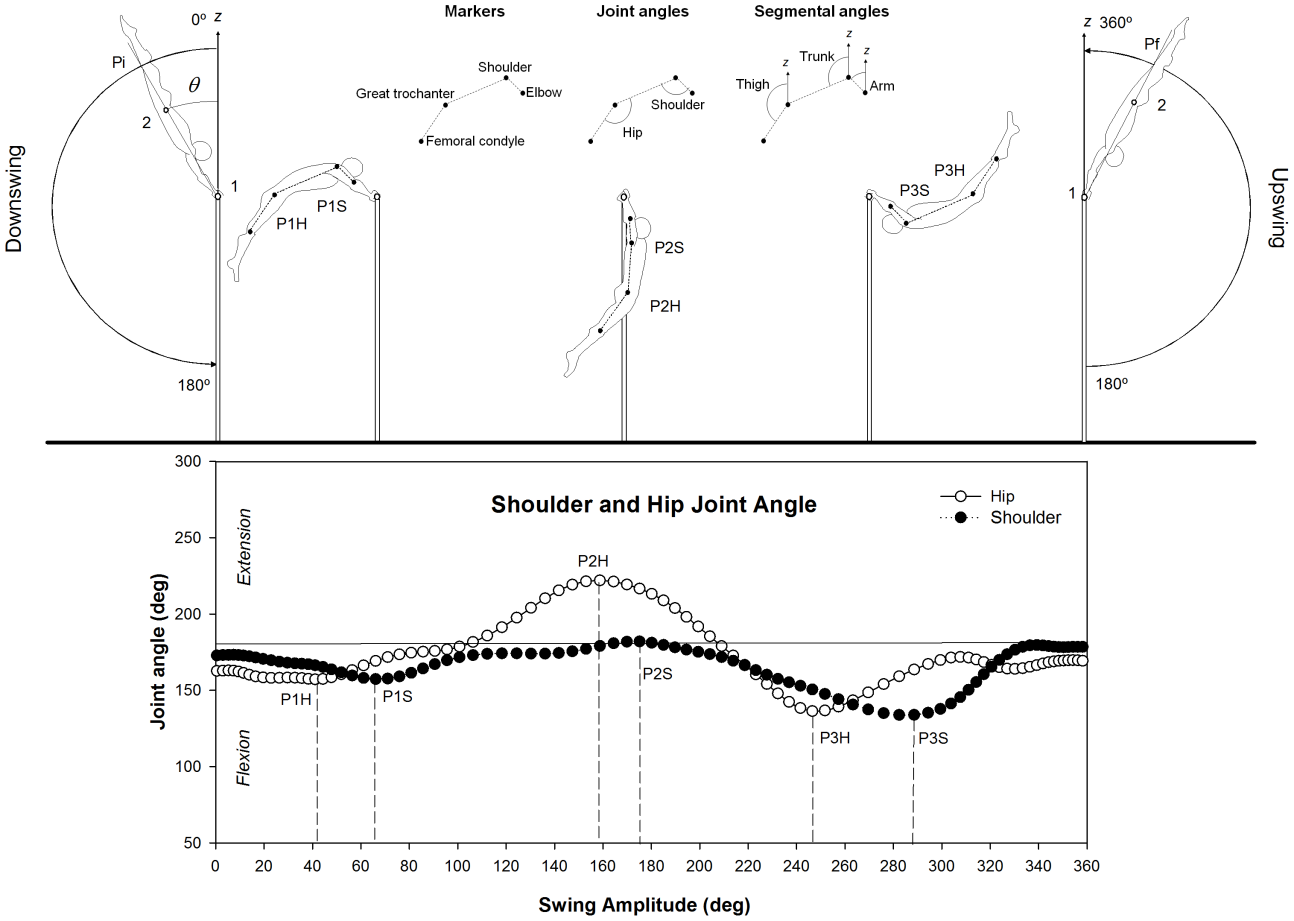
Diagram of the events during a longswing. In the upper section, body position angle (θ) defined by the z axis, middle grasping hand marker (1) and the center of mass (2). Markers (elbow, shoulder, great trochanter and femoral condyle), joint angles (hip and shoulder), and segmental angles (arm, trunk, and thigh) definitions are exemplified. Additionally, we illustrate the initial position (Pi), final position (Pf) and longswing events (P1, P2, and P3) from the hip (H) and shoulder (S) joints. In the lower section, the joint angular displacement of the shoulder and hip during a longswing of an expert gymnast is represented. For simplicity, H and S events have been represented at the same instant of time for P1-P3 in the upper section.

The videotaped images of the longswings were manually digitized by the first author with Ariel Performance Analysis System (APAS System, Inc) and Kwon3D 3.00.033 (Young-Hoo Kwon & Visol, Inc). Raw data were smoothed using Butterworth Low-pass fourth order recursive filter [30]. Cut-off frequency was set at 5 Hz based on a residual analysis and qualitative evaluation of the data [30], [31]. For the sagittal plane, joint flexion-extension angular movements at the hip were derived from the angle between the right thigh and trunk (shoulder, great trochanter and femoral condyle markers, Figure 1) and movements at the shoulder from the angle between the right upper arm and trunk (elbow, shoulder and great trochanter markers, Figure 1). Whereas segmental angles of the arm (shoulder and arm markers), thigh (great trochanter and femoral condyle markers) and trunk (shoulder and great trochanter markers) were calculated relative to the vertical axis (*z*-axis, Figure 1). In addition, a custom software developed in Matlab version 7.01 (Mathworks R14) identified peaks and valleys in the joint angle displacement-time traces to define the events of interest.

Body position angle was defined as the angle formed by the line connecting the center of mass (CM) with the middle of the grasping hand and the vertical (*z*-axis) of the coordinate system [5] (Figure 1). We calculated the location of the CM for novice participants and male gymnasts using Dempster’s [32] and Clauser’s data [33]. The female gymnasts’ CM (average age  =  13.23 years) were computed using Jensen’s equations to subjects between 4 and 20 years [34], [35].

### Task events

Hip (H) and shoulder (S) angle joint movements in the sagittal plane were used to define six events [1], [4], [5] (Figure 1): the minimum angle of the hip and shoulder during downswing (P1H, P1S) and upswing (P3H, P3S), and the maximum angle between P1 and P3 of the hip (P2H) and shoulder (P2S). These hip and shoulder events were expressed in degrees of the complete longswing (i.e., from handstand to handstand).

### Variables

To address the aims of the study, the skill global index was defined as longswing amplitude [28], while coordination was assessed using three types of variables: inter-joint reversal points, positive and negative areas in the continuous relative phase.

Amplitude of a complete longswing was ranged from 0 degrees (vertical position over the high bar before the downswing) to 360 degrees (vertical position over the high bar after the upswing). However, smaller amplitudes than 360° normally occurred during the longswing acquisition. For this reason, we measured the minimum degree of the path (i.e., maximum elevation during the downswing) and the maximum degree of the path (i.e., maximum elevation during the upswing) of each trial in order to define the initial (Pi) and final points (Pf), respectively. The total path between Pi and Pf was the longswing amplitude, our skill global index.

In order to characterize the coordination between the hip and shoulder in the longswing on high bar, we defined inter-joint reversal points (P1H-P1S, P2H-P2S, and P3H-P3S) by subtracting each hip event from its equivalent shoulder event. These inter-joint reversal points indicate the temporality of the hip and shoulder actions and therefore it is a measurement of temporal coordination. Negative values indicate that the shoulder event occurred later than the hip event, while positive values indicate the opposite. Inter-joint reversal points closer to experts’ values indicate better temporal coordination.

Coordination was also assessed with the continuous relative phase (CRP). The CRP was used to represent the phasing relationships or coordination mode between the actions of the two body segments at every point. Although a single CRP between arm and thigh (ATh) could have been defined, we selected to analyse its components (ATh  =  AT + TT) separately to provide congruency with the functional phases (hip and shoulder events) and more direct practical application. Therefore, we calculated arm-trunk (AT) CRP and trunk-thigh (TT) CRP using segmental angular data from arm, trunk, and thigh. Each CRP was obtained by subtracting distal from proximal segmental phase angles [7]. That is, trunk minus thigh for TT and arm minus trunk for AT. The angular displacement (θ) and angular velocity (ω) of each longswing were normalized according to Hamill et al. [22] to allow for the calculation of the phase angles using φ = tan^−1^(ω/θ). In addition, the calculated phase angles were corrected to range values between 0–180° before we computed CRP. Previously defined task events from hip (P1H, P2H, and P3H) and shoulder (P1S, P2S, and P3S) and final position (Pf) were used to divide the arm-trunk (AT) CRP and trunk-thigh (TT) CRP, respectively, to three different phases. To further characterize coordination mode changes, we examined the positive (Pos) and negative (Neg) areas in the continuous relative phase as indicators of the relative angular velocity of the segments. Given that all analyzed body segments move in the same direction during the longswing, changes in the sign of the CRP indicate modifications of the angular velocity relation between two segments. Negative values in the AT CRP mean that the arm moves slower than the trunk, while faster movements of the thigh than the trunk result in negative values in the TT CRP. We compute the positive and negative areas over the interval between events of the same joints: P1H-P2H, P2H-P3H, and P3H-Pf in the trunk-thigh (TT) CRP; and P1S-P2S, P2S-P3S, and P3S-Pf in the arm-trunk (AT) CRP. These positive and negative areas were used to assess differences in coordination mode across groups and practice.

### Statistical Analyses

To address the main purposes of the study we used 2 (Group)×2 (Time) mixed ANOVAs in which group was the between-participants factor and time (first and last trial) the within-participants factor. Post-hoc comparisons between pre- and post-practice within each group were used. To address the secondary goal of this study, one-way ANOVAs with Tukey multiple comparison post hocs were used for establishing differences between the NST, ST and Expert groups at the last trial. Bonferroni’s P adjustments (*p* value x *k* groups) were used to break down the significant 2×2 ANOVA interactions or multiple group comparisons (ST, NST, E) in the post-practice period. Statistical power values were calculated and interpreted as a low (Power <.50), moderate (.50≤ Power <.80) or large (Power ≥.80). The effect sizes were measured with the partial eta squared (η^2^ *p*). The effect size values are considered small (.010≤η^2^ *p*<0.59), medium (0.59≤η^2^ *p*<.138) or large (η^2^ *p*≥.138) [36].

When normal distribution (Kolgomorov-Smirnov Test) and homogeneity of variance (Levene Test) were verified, parametric statistics were used; else rank transformations were used in the 2×2 designs and non-parametric tests were used in the one-way designs.

Statistical significance was set at p<.05 level.Only the statistical significant results were reported. All tests were performed with Systat 11.0 (Systat Software, Inc., San José, CA, USA) and SigmaStat 3.1 (Systat Software, Inc., San José, CA, USA).

## Results

### Non-spontaneously-talented (NST) vs. spontaneously-talented (ST)

**Figure 2:**
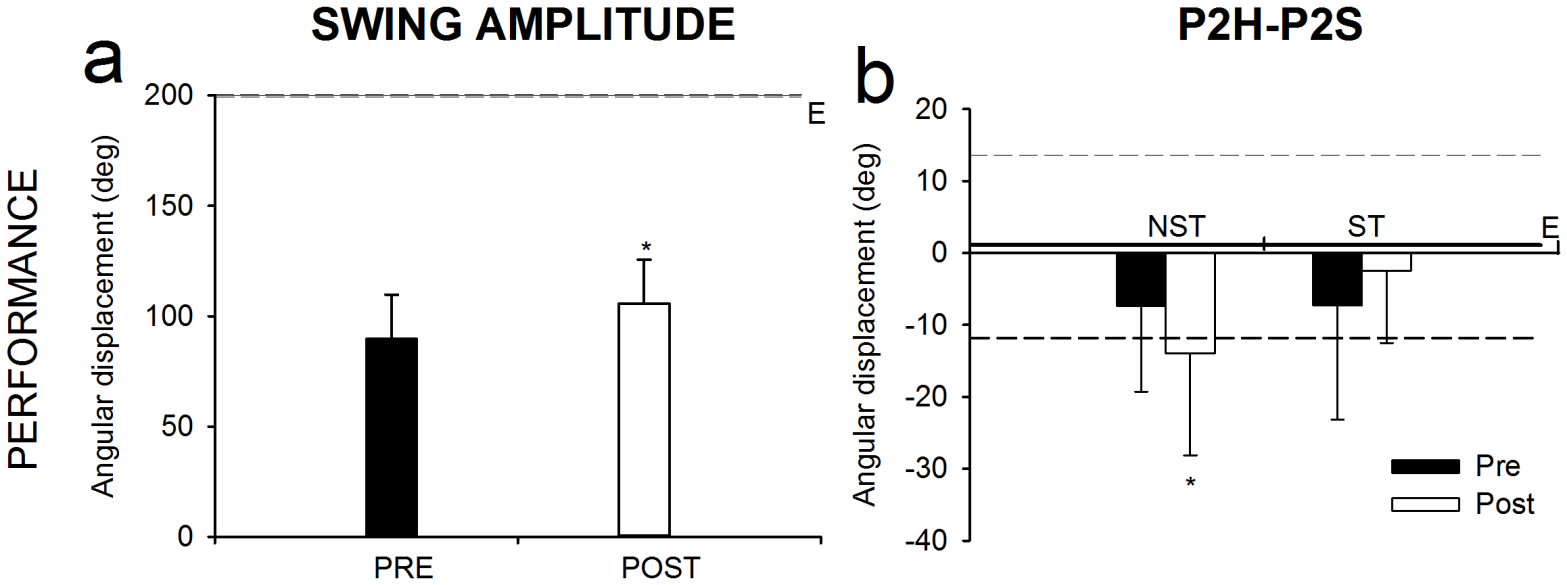
Longswing amplitude and inter-joint reversal point changes after the practice period. Results of the 2 (Group)×2 (Time) ANOVA with repeated measures comparing pre- and post-practice skill global index and coordination variables are depicted in (a) longswing amplitude and (b) inter-joint reversal point P2H-P2S. The expert group (E) mean (solid line) and standard deviation (dashed lines) of these variables are provided for comparison. (★) indicates significant group or time differences. Interaction effects are not presented for simplicity.

Longswing amplitude was larger in the ST than the NST group and practice improved amplitude in both groups (Figure 2a). The 2×2 ANOVA for the longswing amplitude revealed significant group and trial (time) main effects (Table 1).

**Table 1:** Significant results of two (Group) x two (Time) ANOVA with repeated measures (RM).

		2×2 ANOVA RM					
Variable group	Variable name	Main effects and interaction	F	Degrees of freedom	p	Power	η*^2^*p
	Longswing Amplitude	Group	10.28	1,22	.004	.87	.696
		Time	26.38	1,49	.000	.99	.590
*Longswing Inter-joint Reversal Points*	P2H-P2S	Group x Time	5.09	1,22	.034	.58	.181
*Positive Areas in CRP*	AT-Pos P2S-P3S	Group	4.70	1,22	.041	.44	.283
		Time	7.46	1,49	.012	.69	.245
	AT-Pos P3S-Pf ^a^	Group	6.64	1.22	.017	.62	.367
*Negative Areas in CRP*	TT-Neg P1H-P2H	Time	7.95	1,49	.010	.72	.257

P1, P2, and P3 represent hip (H) and shoulder (S) events, while Pf stands for the final position. CRP = continuous relative phase; Pos = positive area; Neg = negative area.

a Test of normality failed. Values were transformed on ranks to compute ANOVA.

The timing of hip and shoulder actions in maximum extension events (i.e., inter-joint reversal point for P2H-P2S) showed that the NST group maintained P2H-P2S similar values from pre- to post-practice while the ST group had a tendency to improve (becoming closer to reference values, in this case values of the expert group, Figure 2b). The analysis of the 2×2 ANOVA for the inter-joint reversal point for P2H-P2S yielded a significant ‘group by time’ interaction with a large effect size and moderate power (Table 1). However, statistics did not show significant simple main effects.

**Figure 3:**
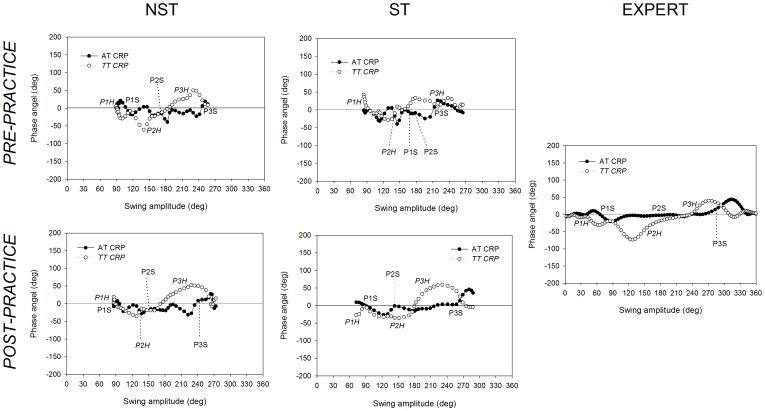
Continuous relative phases obtained from one participant in each group before and after the practice. Representative continuous relative phases (CRP) of the arm-trunk (AT) and trunk-thigh (TT) coordination obtained from one participant in each novice group (spontaneously-talented, ST, and non-spontaneously-talented, NST) and expert group when they performed a longswing on high bar. CRPs acquired before and after the practice period (pre- and post-practice, respectively) are depicted for the ST and NST groups.

Arm-trunk (AT) and trunk-thigh (TT) continuous relative phase (CRP) graphs for a longswing on high bar are plotted in Figure 3. These graphs show representative examples from novice groups (ST and NST) before and after the period of practice (pre- and post-practice). CRP curve changes from pre- to post-practice represent modifications in the coordination mode of the two analyzed body segments.

**Figure 4:**
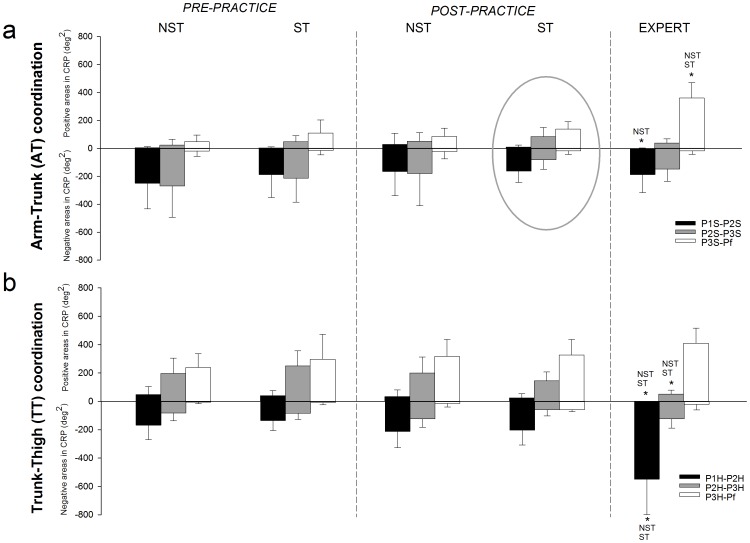
Positive and negative areas in the continuous relative phase for each group. Positive and negative areas’ mean and standard deviation for each group (spontaneously-talented, ST, non-spontaneously-talented, NST, and experts) are depicted in: (a) arm-trunk (AT) coordination and (b) trunk-thigh (TT) coordination. Positive and negative areas are calculated over the interval between events of the same joints: P1S-P2S, P2S-P3S, and P3S-Pf in the AT continuous relative phase; and P1H-P2H, P2H-P3H, and P3H-Pf in the TT continuous relative phase. AT coordination mode for the ST during post-practice is highlighted by a circle in order to emphasize the progression toward the expert coordination mode. (★) indicates significant group differences comparing novice (NST and ST) post-practice and experts.

Figure 4 depicts the arm-trunk (AT) and trunk-thigh (TT) coordination modes as positive and negative areas of the continuous relative phase (CRP) between events. Areas represent the relative angular velocities of the two body segments during these intervals of the longswing limited by the consecutive events of the same joint. From maximum flexion during downswing to maximum extension (P1–P2), changes were observed for both the arm-trunk (AT) and trunk-thigh (TT) coordination modes. For the AT coordination, the NST group seemed to enlarge the arm angular velocity in relation to the trunk (positive area) while the ST did not change this relative velocity. However, the 2×2 ANOVA did not present significant results for this variable. It is important to note that NST group in arm-trunk (AT) positive and negative area showed more variability between participants (i.e., standard deviation, Figure 4a). For the trunk-thigh (TT) coordination, the negative area between hip maximum flexion during downswing and maximum extension (P1H-P2H) increased due to practice taken both groups together (Figure 4b), suggesting that the thigh angular velocity relative to the trunk increased as a result of practice. The 2×2 ANOVA resulted in a significant time main effect with a large effect size and a moderate power for trunk-thigh negative area delimited by hip maximum flexion during downswing and maximum extension (P1H-P2H) (Table 1).

The arm-trunk relative angular velocity from shoulder maximum extension to final position (i.e., AT-Pos P2S-P3S and AT-Pos P3S-Pf) demonstrated greater velocity for the arm in relation to the trunk (positive area) for the ST group than NST considering both times (pre- and post-practice) together. In addition, arm-trunk positive areas between shoulder maximum extension to maximum flexion during upswing (AT-Pos P2S-P3S) was also impacted by practice, that is, the relative angular velocity of the arm in relation to the trunk increased taken both groups together. The 2×2 ANOVA yielded significant group main effects for the AT-Pos P2S-P3S and the AT-Pos P3S-Pf, and a time main effect for the AT-Pos P2S-P3S (Table 1). Effect sizes were large for these three main effects; however, power was low for the AT-Pos P2S-P3S group main effect while AT-Pos P3S-Pf group and AT-Pos P2S-P3S time main effects presented moderate power.

### Novices vs. Experts (3 groups)

Examining group differences at post-practice, the expert group demonstrated larger longswing amplitude than both novices group. The One-way ANOVA was significant (F_2,30_  =  28.84, p = .000, η^2^ *p* = .650). For the coordination variables (i.e., longswing inter-joints reversal points, and positive and negative areas in CRP) the expert group was also different from novices. These differences can be observed qualitatively in the CRP curves presented in Figure 3.

Regarding the inter-joint reversal point, ST presented closer values to reference values form experts than NST. Despite the One-Way ANOVA yielded significant differences between the three groups for inter-joint reversal point between hip and shoulder maximum extension (P2H-P2S), significant simple main effects were not found in the P2H-P2S. When examining the positive areas from the shoulder maximum flexion during downswing (AT-Pos P1S-P2S) to final position (AT-Pos P3S-Pf), the expert group showed a progression from low relative angular velocity between arm and trunk to a faster movement of the arm in relation to the trunk. However, the NST group demonstrated larger relative velocity of the arm between shoulder maximum flexion during downswing and maximum extension (P1S-P2S) than the Expert group (Figure 4a). In addition, Expert group moved the arm significantly faster in relation to the trunk than novice groups from shoulder maximum flexion during upswing to final position (P3S-Pf, Figure 4a). A similar progression in trunk-thigh (TT) coordination mode was observed for the expert group from hip maximum flexion during downswing to final position (i.e., TT-Pos P1H-P2H to TT-Pos P3H-Pf) where positive areas were almost negligible initially, but trunk acquired a faster angular movement in relation to the thigh towards the end of the longswing. ANOVAs analysis yielded significant smaller trunk velocities in relation to the thigh from hip maximum flexion during downswing to maximum extension (P1H-P2H) and from hip maximum extension to maximum flexion during upswing (P2H-P3H) for the Expert group (Figure 4b). When examining the negative areas, none of the variables showed significant differences between groups except for experts showing larger relative velocity of the thigh compared to the trunk between hip maximum flexion during downswing and maximum extension (TT-Neg P1H-P2H) than the novice groups (Figure 4b).

## Discussion

The main aim of this study was to describe coordination changes in the acquisition of a novel task (longswing on high bar) due to practice and initial talent. We hypothesized larger improvements in coordination for the spontaneously-talented group (ST) after similar content and amount of practice than the non-spontaneously-talented group (NST). The novice participants’ longswing amplitudes (i.e., skill global index) increased as a result of a two-month practice period. The skill global index improved to a similar extent in both groups (NST group improved 18% of the longswing amplitude and ST group improved 11%) suggesting a comparable effect of practice. Improvements after practice for oscillation amplitude in novice participants were also found by several authors in different swing skills: in parallel bars [19], lateral swing on a suspended platform [20], and swing on a ski apparatus [37]. These authors proposed that improvements in skill global index due to practice in novice participants may not necessarily imply changes in coordination. Their findings could be explained on the theoretical basis that performance and coordination may have different acquisition time rates [24], [25]: first, early learners improve the task execution due to performance acquisition (i.e., events placement), later, skilled movements will demonstrate improved coordination. In this study, we explored how each group accomplishes this improvement via potential changes in their inter-segmental coordination, as well as to observe whether changes in both groups are closer to an effective coordination mode (expert gymnasts’ coordination for the purpose of this paper).

While our skill global index (longswing amplitude) did not present differences between novices groups, coordination variables (the inter-joint reversal points and positive area in the continuous relative phase) suggested differential group effects due to practice. In fact, the inter-joint reversal points for the hip and shoulder maximum extension (P2H-P2S) critically differentiated the two groups. At the beginning of the study novice groups showed similar values for this variable. The ST group reduced the time lag between the events of hip and shoulder extension during the downswing (0–180°) getting them closer to expert values. The NST group changed P2H-P2S inter-joint reversal point (farther from expert values), suggesting that these two events (P2H and P2S) occurred more distant from each other in time, despite that at the beginning of the study they showed similar values to the ST group. These improvements in the inter-joint reversal points for the participants with better initial skill level occurred along with a coordination mode significantly closer to experts’ coordination, especially in the arm-trunk (AT) coordination mode. The ST group progressed from a low arm-trunk positive relative angular velocity during the initial part of the longswing (P1S-P2S, from shoulder maximum flexion during downswing to maximum extension) to a faster arm movement in relation to the trunk in the final part (P3S-Pf, from shoulder maximum flexion during upswing to final position). This progression made ST group coordination mode closer to that of the expert group. Opposite to the experts, and to achieve this progression, the ST group modified coordination between shoulder maximum extension and maximum flexion during upswing (P2S-P3S) increasing the positive and decreasing the negative areas. Indeed, the ST group moved the arm faster in relation to the trunk in P2S-P3S than the NST and expert groups suggesting that spontaneously-talented participants resorted to a shoulder joint action to accomplish better performance.

These results seem to support other studies that divided new task acquisition in two stages [24], [25]: (1) appropriate placement of task events, that is, the spatial sequences of the movement, and (2) the dynamic control of the action. We would suggest that only the ST group entered into the second acquisition stage by the end of practice, given that they were able to modify their coordination. More concretely, the ST group acquired near expert spatial sequence (i.e., inter-joint reversal points) while, as a result of practice, the coordination mode between segments was still approaching that of experts. In addition, given these results, we would like to propose that the time rate to improve coordination after a similar amount of practice is affected not only by task difficulty and complexity, as proposed by Teulier et al. [37] in their swing in ski simulator and Delignières et al. [19] in parallel bars studies, but also by the initial skill level of the performer.

On the other hand, the expert coordination mode was different to the novice participants in two aspects. First, the experts showed a progressive increment of positive area in the CRP along the whole longswing. Second, we observed a large amount of the negative area between hip maximum flexion during downswing and maximum extension (P1H-P2H) in the experts located before the hip extension. Both novices group, ST and NST, enlarged the negative area in P1H-P2H due to practice, but post-practice values of this variable were still far away from the expert values. We interpret these large experts’ thigh velocities during the P1H-P2H phase as a preparative but necessary action to accomplish the upcoming maximum extension of the hip at P2. Based on the existing literature, including coaching and scientific based data [5], [6], we expected a trunk-thigh (TT) continuous relative phase (CRP) curve with a single negative peak between hip maximum flexion during downswing (P1H) and hip maximum extension (P2H), given that the expert gymnasts produce a single hip extension action within these two events. Surprisingly, a double negative peak curve was found in the Expert group in this period of the longswing. We interpret these results as an indication that expert mode of coordination included two preparatory actions instead of the single one previously proposed in the literature. Given the relative small sample and the training homogeneity of expert gymnast group used in this study, further studies with more participants and with different gymnastics background will be needed to confirm these findings.

Although the complex process of learning cannot be characterized, our study study design let us to describe the coordination changes occurred from the initial to the last session. On the basis of the experimenters’ experiences in coaching gymnasts, coordination changes described in this study could be applicable to other groups and ages that are faced with a longswing as a novel task. This extrapolation, however, needs to be made with caution due to the moderate power values achieved. Given the large differences (i.e., large effect size) found in all variables with significant results, it would be possible that power improve with a larger sample in each group. We believe that different levels of longswing amplitude and expertise will be related with specific coordination modes. However, not only further research is necessary to support this claim, but also to assess the complete learning process. To achieve this goal, it would be necessary to analyze every trial in each training session.

## Conclusions

Our findings have shown that novice participants improved longswing amplitude with practice, but the coordination in the downswing was different for the ST and the NST after practice. It seems as if the ST group was able to modify and improve the temporal aspect of the coordination (inter-joint reversal points) while the dynamic aspects of the coordination (coordination mode) were not totally acquired yet. However, and despite of the initial skill level, upswing coordination will require more focused practice, especially for the arm-trunk coordination. We propose to focus practice in the following 3 steps: (1) focus on the placement of the downswing hip and shoulder events, (2) improve the coordination mode of the shoulder events in the downswing, and (3) develop better coordination in the upswing.
